# Forest understory vegetation study: current status and future trends

**DOI:** 10.48130/FR-2023-0006

**Published:** 2023-03-14

**Authors:** Jiaojiao Deng, Shuai Fang, Xiangmin Fang, Yanqiang Jin, Yuanwen Kuang, Fangmei Lin, Jiaqing Liu, Jingran Ma, Yanxia Nie, Shengnan Ouyang, Jing Ren, Liehua Tie, Songbo Tang, Xiangping Tan, Xugao Wang, Zhaofei Fan, Qing-Wei Wang, Hang Wang, Chenggang Liu

**Affiliations:** 1 CAS Key Laboratory of Forest Ecology and Management, Institute of Applied Ecology, Chinese Academy of Sciences, Shenyang 110016, China; 2 College of Forestry, Jiangxi Agricultural University, Nanchang 330045, China; 3 CAS Key Laboratory of Tropical Plant Resources and Sustainable Use, Xishuangbanna Tropical Botanical Garden, Chinese Academy of Sciences, Menglun 666303, China; 4 Yuanjiang Savanna Ecosystem Research Station, Xishuangbanna Tropical Botanical Garden, Chinese Academy of Sciences, Yuanjiang 653300, China; 5 Key Laboratory of Vegetation Restoration and Management of Degraded Ecosystem, South China Botanical Garden, Chinese Academy of Sciences, Guangzhou 510650, China; 6 University of Chinese Academy of Sciences, Beijing 100049, China; 7 College of Ecology and Environment, Xinjiang University, Urumqi 830000, China; 8 Institute for Forest Resources and Environment of Guizhou, Key Laboratory of Forest Cultivation in Plateau Mountain of Guizhou Province, College of Forestry, Guizhou University, Guiyang 550025, China; 9 School of Ecological and Environmental Sciences, East China Normal University, Shanghai 200241, China; 10 School of Forestry and Wildlife Science, Auburn University, AL 36830, United States; 11 Yunnan Key Laboratory of Plateau Wetland Conservation, Restoration and Ecological Services, National Plateau Wetlands Research Center, Southwest Forestry University, Kunming 650224, China

**Keywords:** Biodiversity, Functional traits, Forest regeneration, Biomass modeling, Carbon sequestration, Nutrient cycling, Multiple functions, Forest management practice, Global change.

## Abstract

Understory vegetation accounts for a large proportion of floral diversity. It provides various ecosystem functions and services, such as productivity, nutrient cycling, organic matter decomposition and ecosystem self-regeneration. This review summarizes the available literature on the current status and progress of the ten most studied branches of understory vegetation on both its structural and functional aspects based on global climate change and forest management practices. Future research directions and priorities for each branch is suggested, where understory vegetation in response to the interplay of multiple environmental factors and its long-term monitoring using ground-based surveys combined with more efficient modern techniques is highlighted, although the critical role of understory vegetation in ecosystem processes individually verified in the context of management practices or climate changes have been extensively investigated. In summary, this review provides insights into the effective management of the regeneration and restoration of forest ecosystems, as well as the maintenance of ecosystem multilevel structures, spatial patterns, and ecological functions.

## Introduction

**Figure 1:**
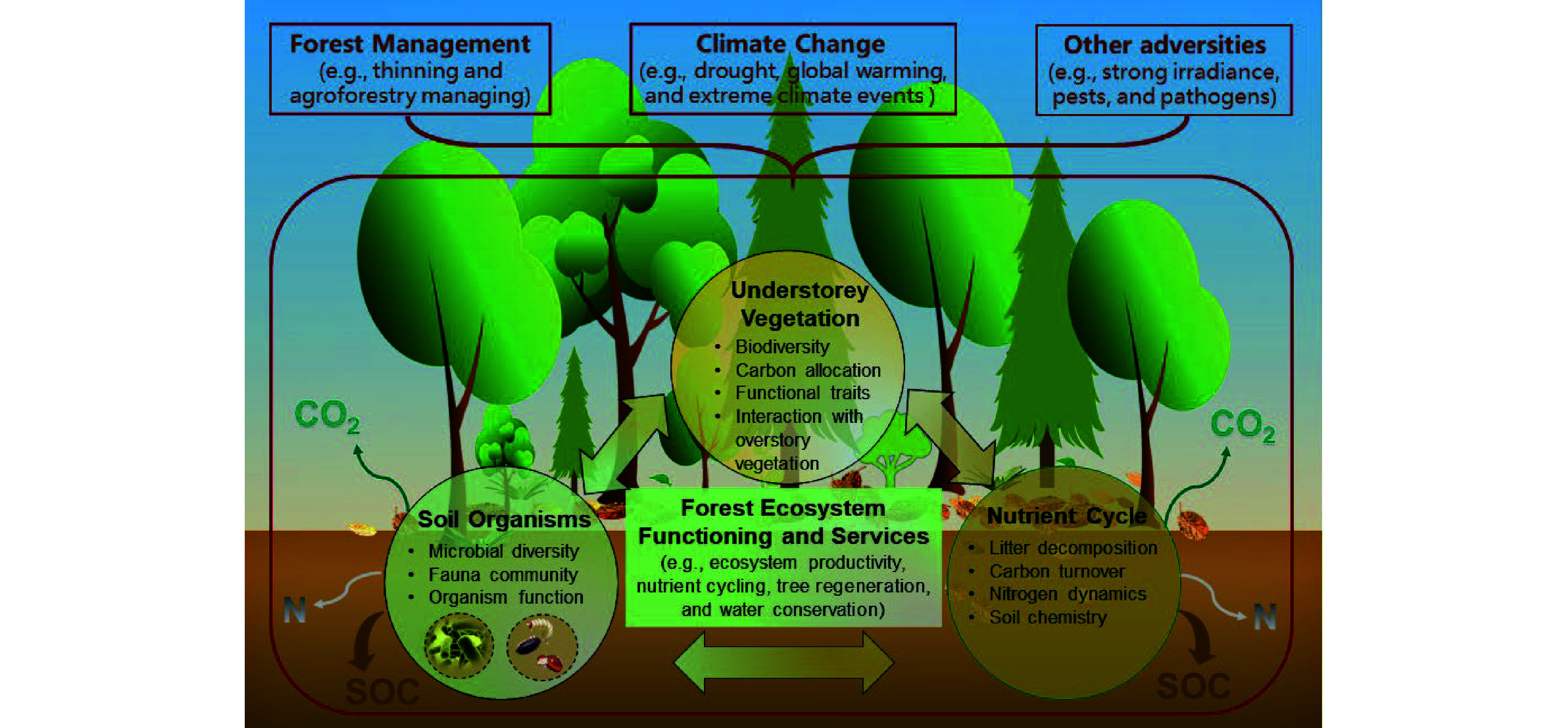
Conceptual roles of understory vegetation in forest ecosystem functioning and services, and its response to management practice and climate change. CO_2_, carbon dioxide; SOC, soil organic carbon; N, nitrogen.

Understory vegetation is a vital stratum of forests, including seedlings (< 1.5 m tall), shrubs, herbs, bryoids (i.e., mosses and lichens), and lianas. Both vascular (woody and non-woody) and nonvascular (liverworts, hornworts, and mosses) plants are essential components of understory vegetation^[1]^, which plays important roles in forest ecosystem structure and functioning (e.g., biodiversity, regeneration, biomass, and nutrient content, as well as functional traits) and provision of ecological services (e.g., soil nutrient cycling and biological processes, water conservation, as well as mitigating emissions of greenhouse gas from carbon (C) sequestration and nitrogen (N) dynamics) (Fig. 1)^[2,3]^. They can also provide food, shelter, and habitat for animal species, especially for soil arthropods and large herbivores^[4]^. Nevertheless, the composition and distribution of understory vegetation are strongly influenced by competition (generalizability) or facilitation (specificity) from the overstory trees^[5−7]^. Understory species are always seen as 'noxious weeds' with exploitative competition for light, water, and nutrients or interference competition involving allelopathic effects against target overstory trees, especially plantation forests^[8,9]^. Moreover, overstory management, mainly thinning and pruning practices, can directly and indirectly affect the formation of understory vegetation, which in turn regulates understory community resistance, adaptation, and resilience to general climate changes (e.g., drought, global warming, and N deposition), extreme climate events (e.g., fire, heatwaves, and freezing rain), and other adversities (e.g., strong irradiance, pests, and pathogens)^[10−12]^. As a result, more experiments based on understory removal and overstory management are being carried out to explore the role of understory vegetation in ecosystem function, with some controversial results^[9,13,14]^. This review recapitulated the current status, progress, and future direction of forest understory vegetation studies. We expect that this review can help understand the mechanisms of understory developments to support ecosystem multifunctionality and sustainability against unpredictable global climate change. We hope that it will also provide professionals, managers, and policy makers with scientific guidelines for the sustainable management of forests.

## Biodiversity of understory vegetation

High diversity is vital for maintaining multiple ecosystem functions^[15]^ and regulating forest regeneration dynamics^[16]^. Understory vegetation biodiversity often exhibits significant variations along a range of abiotic and biotic gradients (e.g., rainfall^[17]^, productivity^[18]^, and latitude^[19]^), contributing to the structural complexity and biodiversity of forest ecosystems^[20,21]^. In tropical forests, herbaceous plants generally account for 14%–40% of all forest vascular species^[22]^, in contrast to more than 80% of vascular species in temperate forests^[20]^. However, lianas and epiphytes account for 27% of plant species in tropical forests, but they are depauperate in temperate forests (< 3% of species). The proportion of shrubs in tropical forests is also 7% higher than in temperate forests^[16]^. Spatial and temporal variations in the biodiversity of understory vegetation can lead to significant differences in forest ecosystem functioning^[15]^. Therefore, understanding the driving mechanisms of understory biodiversity can have important implications for biodiversity conservation and ecosystem function maintenance in global climate change.

**Figure 2:**
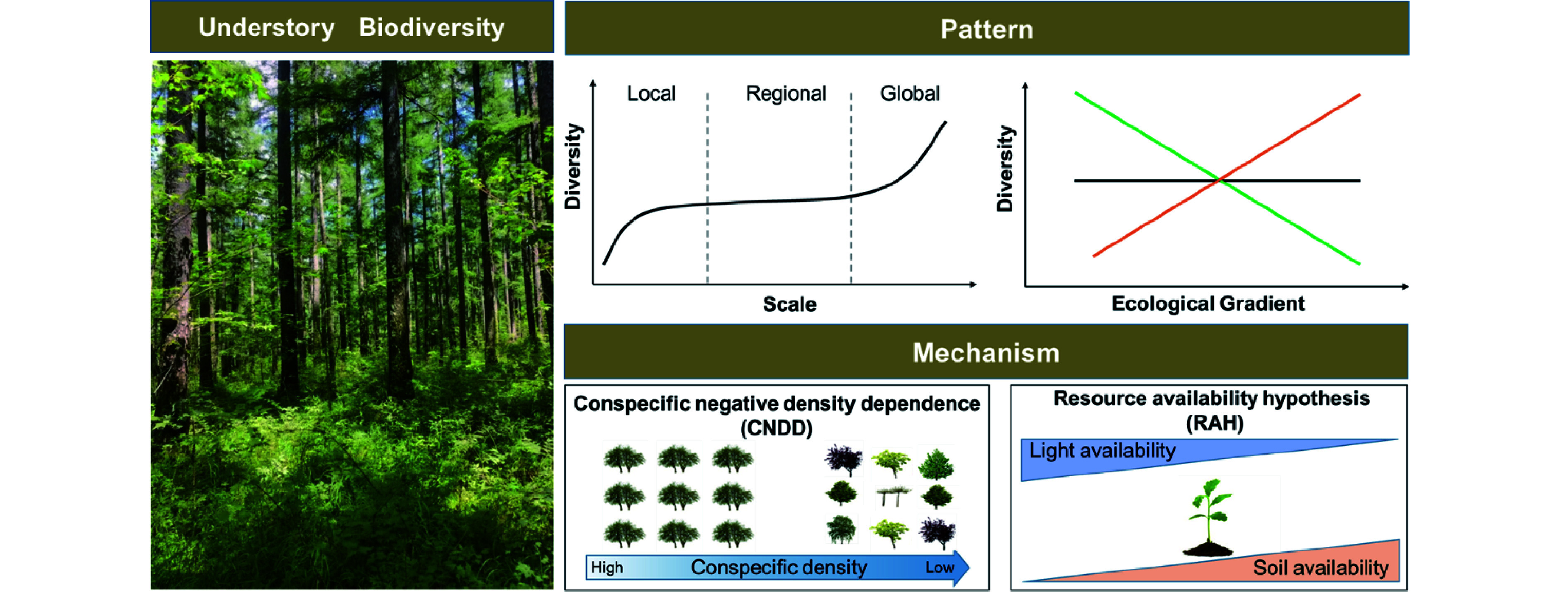
The patterns and mechanisms underlying understory vegetation diversity. 1) Understory diversity pattern varies significantly along spatial scales and ecological gradients. 2) Conspecific negative density dependence (CNDD) and resource availability hypothesis (RAH), which can be used to account for the formation and maintenance mechanisms of understory vegetation biodiversity. The strength of CNDD change along the conspecific density gradient, while the role of RAH varies with resource (e.g., light and soil fertility) availability.

Various mechanisms have been proposed to account for the formation and maintenance mechanisms of understory vegetation biodiversity^[23−26]^. Though they are far from conclusive, there are two dominant hypotheses in the context of intraspecific competition and resource availability (Fig. 2). Conspecific negative density dependence (CNDD), which is also known as the Janzen-Connell hypothesis (JCH), is a critical explanation^[27,28]^. It assumes that the offspring's demography performance (e.g., growth, recruitment, and survival) would be reduced when surrounded by a higher density of conspecific adults or located near conspecific adult trees due to similar resources requirements or through host-specific enemy attacks^[27,28]^. As a result, CNDD is generally stronger for common species than for rare ones, thus regulating the understory diversity. Numerous studies have investigated the effects of CNDD in driving understory species diversity^[29−32]^, but results show that its strength varies significantly among climatic zones^[33−35]^ and local habitat conditions^[36,37]^. In particular, the effect of CNDD increases with increasing resource availability^[36,37]^ and from the temperate to tropical zones^[33−35]^. Plant functional groups also show different susceptibilities to CNDD^[38−40]^; fast-growing species, arbuscular mycorrhizal species, and shade-tolerant species suffer stronger CNDD than slow-growing species, ectomycorrhizal species, and shade-intolerant species, respectively. In addition, increasing mechanistic studies have shown that other biotic stress (e.g., fungal pathogens, insects, and herbivores) is likely to be a driving force behind these patterns^[41−43]^.

The resource availability hypothesis (RAH) is another dominant interpretation for variability in understory vegetation diversity since understory vegetation inhabits resource-limited environments^[44,45]^. RAH highlights the role of environmental or resource heterogeneity (e.g., light and soil nutrients). For example, increased light availability not only promotes the growth and survival of understory vegetation, but also provides additional ecological niches for species with different life history strategies to meet their light demand, thereby increasing diversity^[46−48]^. Soil properties are also critical factors driving understory species diversity^[49,50]^. The diversity of understory species decreases with decreasing soil pH and available N and phosphorus (P) contents. Moreover, succession history^[51]^, forest management strategies, and fragmentation^[52]^ can also drive understory vegetation diversity by altering the supply of resources. Over the last decades, evidence has accumulated that there are persistent imprints on understory vegetation diversity^[53]^ by anthropogenic disturbances, e.g., thinning^[10]^, invasive species^[54]^, and wildfires^[55]^, as well as climate change, e.g., drought^[56]^, warming^[57]^, and acidifying deposition^[58]^.

Understanding the patterns and mechanisms underlying understory vegetation biodiversity is a longstanding goal in ecology. However, there are still significant gaps ranging from the lack of integrated studies considering multiple climatic factors, neglecting the interaction of multiple trophic levels, and the lack of practical monitoring tools. Hence, we devise three directions for future research. First, climate change has been widely recognized as a major driver of species diversity. Multiple climate variables can interactively influence plant diversity at local, regional, and global scales. Because most previous studies focused on individual climate factors, it is difficult to reflect the actual situation under climate change. Therefore, it is necessary to strengthen the study on the mechanisms of understory vegetation responding to the interaction of multiple climate factors. Second, the high diversity enables the understory to be a 'biodiversity reservoir' and to provide better habitat and refuge for arthropods and large herbivores^[4]^. For instance, large herbivores are critical factors that maintain understory diversity by consuming vegetation. Herbivores can also alleviate plant competition for light by selectively targeting taller species and increasing the availability of light for shorter plants^[59]^. However, it remains unclear how multiple trophic interactions contribute to maintaining species abundance and richness of understory vegetation and ecosystem function. Third, accurate and more efficient characterization and measurement of understory species diversity in the context of global climate change and biodiversity loss remains an issue. Currently, the monitoring of understory vegetation is still dominated by ground-based surveys. Although this approach can accurately describe the vegetation composition and dynamics, it is time-consuming and laborious at larger scales and over extended periods. Application of more efficient methods, such as 3D LiDAR scanning technology and hyperspectral scanning technology, would greatly improve the understanding of understory vegetation diversity.

## Regeneration of understory vegetation

**Figure 3:**
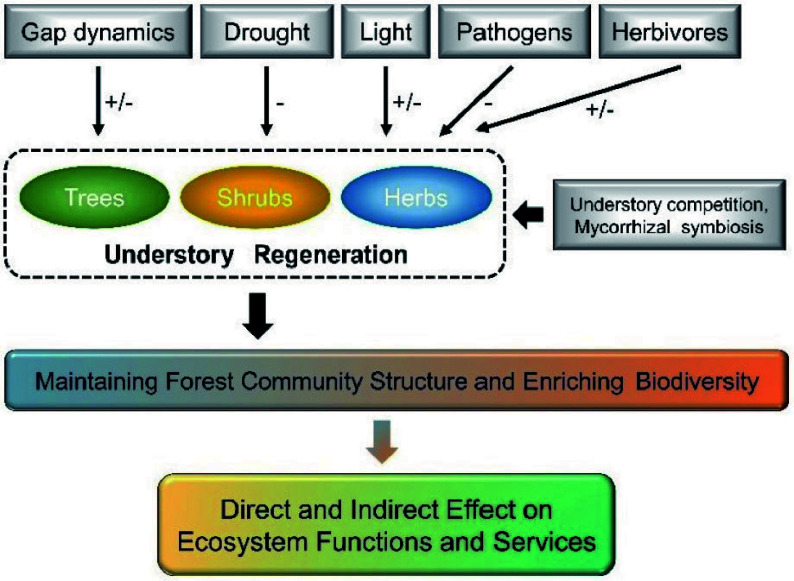
Pathways of bio-abiotic factors effect on understory vegetation regeneration. Positive effects of bio-abiotic factors to understory vegetation are shown as plus signs (+), and negative effects as negative signs (−).

Understory vegetation regeneration is a crucial ecological process in forest ecosystems to achieve self-reproduction and recovery, which is essential for maintaining forest community structure and enriching biodiversity^[60]^. Understory regeneration includes trees, shrubs, herbs, and bryophytes, with tree regeneration being the dominant process in forest understory layer. It also involves the various stages of plant growth from seed production, dispersal, and germination to seedlings' settlement, survival, and growth^[61]^. The seedling stage is most sensitive to external environments during tree regeneration^[62]^, which is particularly vulnerable to abiotic factors (e.g., gap dynamics, drought, and shading) and more susceptible to stress from pathogens and herbivores^[63,64]^, as well as other biological factors (e.g., competition from neighboring plants and effect by mycorrhizal fungi symbiosis) (Fig. 3)^[3,65,66]^. Forest gap disturbance and understory vegetation competition are key bio-abiotic effect factors of primary forest regeneration processes^[3,67,68]^. Forest gaps can adjust the competition between tree seedlings and understory plants by changing stand structure and microenvironmental factors (including sunlight, moisture, and soil nutrients)^[69−72]^. Among these abiotic and biotic factors, sunlight is widely accepted as the dominating factor driving understory regeneration since it provides vital energy and signals for plant grow and development and is the most heterogenous factor in forests^[73−75]^. Here, we reviewed the unique role of sunlight (light intensity and light quality) concerning understory vegetation regeneration and discussed its drivers.

Sunlight can strongly influence the survival and growth of understory vegetation. Its effect magnitude may depend on habitat light intensity^[76]^. Light availability substantially impacts understory shrub and herb communities by changing vital functional traits, mainly due to responses by different species groups^[47]^. Low light availability in the understory is generally considered a stress for tree seedlings, limiting their net C accumulation and growth^[14]^, while high light intensity can increase their growth rates. Thus, light heterogeneity may determine understory vegetation biodiversity due to differences in light utilization strategies among species^[77]^. More critically, solar radiation penetrating the understory has substantial seasonal and diurnal variability in the spectral composition (from 280 to 750 nm, including ultraviolet radiation, visible light, and far-red light). Such variation is caused by changes in solar zenith angle, wind disturbance, and forest canopy effects (e.g. light attenuation, absorption, and reflection by overstory leaves)^[78]^. Molecular studies on model species find that each spectral composition can specifically regulate a wide range of plant ecophysiological processes (seed generation, leaf photosynthesis, morphology, and biomass accumulation), individual performance, growth, and phenology^[79−81]^. Recent studies on understory herbaceous plants have found that ultraviolet-B (UV-B) radiation (280−315 nm) significantly reduces the total leaf area of shade-tolerant species, while blue light (400−500 nm) significantly increase their photosynthetic rate^[82]^. For tree species with a long-life cycle, previous studies involving spectral interaction (light quality) mainly focus on the ratio of red light to far-red light (R/FR) in the context of shade syndrome^[83−85]^. For instance, Razzak et al.^[86]^ found that a high R/FR ratio significantly increased the cotyledon length and chlorophyll content of Scottish pine seedlings, while a low ratio increased the hypocotyl length. In terms of canopy spectral biology, however, most results are obtained in the laboratory or greenhouse at fixed spectral ratios of the visible bands. At the same time, light effects on tree seedlings are still less clear.

Recently, a few studies have attempted to explore how changes in canopy spectral composition regulate tree seeding growth, aiming to provide new scope to forest regeneration. For instance, one growth-chamber study using LED spectral control indicated that blue light increased the sturdiness and altered the branching patterns in seedlings of Norway spruce and Scots pine^[87]^ while improving the water-use efficiency (WUE) of silver birch^[88]^. Another garden study found that seedlings of *Pinus koraiensis* and *Quercus mongolica* had significantly different spectral adaptation strategies^[89]^. *Q. mongolica* tends to use spectral changes to adjust morphology to increase light capture ability, while *P. koraiensis* adjusted physiological and biochemical processes to improve C assimilation efficiency. Such results are consistent with those from shade-tolerant and intolerant herbs in the understory^[82,90]^. However, the lack of studies on tree species to solar radiation limits our exploration of the mechanism of light-driven forest regeneration.

Global changes will profoundly affect future forest ecosystems^[91]^, driving understory vegetation regeneration and understory functioning by altering resource availability and habitats. Extreme climate events are projected to become more prevalent in the future and significantly modify solar spectral composition (e.g., evaluating solar UV radiation) that understory tree species receive, by changing atmospheric conditions (e.g., stratospheric ozone, aerosols, and cloud cover), modification of land cover (e.g., snow, ice, and vegetation), and alteration in the timing of development in organisms (i.e., phenology)^[92]^. However, there are still significant uncertainties about how understory regeneration is affected by the interaction of global change and solar radiation. To better understand the underlying mechanism of understory regeneration, we suggested focusing on the following aspects: First, direct and indirect effects *via* global changes will be equally crucial in determining understory regeneration, especially plant species' light competition. Few studies focus on the mutual effect of understory solar spectral composition and N deposition on tree regeneration. Second, understory plants typically grow in dynamic natural light environments^[93,94]^. However, most existing studies have focused on seedlings' growth under constant light conditions, which may over or underestimate the effect of fluctuating light on seedling growth. Therefore, evaluating seedlings' responses under varying light conditions is critical. Third, studies of how light intervenes plant–soil feedbacks and affects understory vegetation regeneration are still rare. Incorporating the above- and below-ground responses of seedlings into a unified framework in the context of global change will provide novel insights for understory regeneration.

## Biomass, nutrient content and storage of understory vegetation

Assessment of biomass storage is often an essential part of quantifying nutrient storage. Biomass storage is generally used to evaluate the performance and health of different plant individuals or strata in forest ecosystems. Variations in forest biomass and nutrient storage have been well studied across different forest types and spatial-temporal scales^[95−97]^, while these studies usually exclude understory vegetation. Understory plays a crucial role in nutrient cycling and maintaining forest productivity. Therefore, understory biomass and nutrient storage have recently gained more attention. This section will review the understory vegetation biomass and its contribution to the nutrient storage of forest ecosystems.

As an essential component of a forest ecosystem, understory species contribute disproportionately to forest biomass^[98]^. Understory vegetation biomass averaged 6.5 t·ha^–1^, contributing more than 6% to total forest biomass^[99]^. It varied largely with climatic regions and forest types. The average understory biomass in the cold temperate region is higher than in other climate regions. The average understory biomass is higher in natural forests than in planted forests at a large scale^[99]^. Also, the changes in understory biomass show an apparent spatial pattern^[99]^. Specifically, understory biomass decreased with increasing longitude but decreasing latitude, elevation, and mean annual temperature and precipitation. These findings provide a comprehensive understanding of the spatial distribution pattern of understory biomass. These spatial patterns may be attributable to the variation of canopy tree species composition and environmental conditions (e.g., precipitation, light, temperature, soil, and topography), which in turn could alter resource distribution in the forest understory and then affect the establishment of understory plants and understory biomass accumulation. Stand structure strongly affects the changes in understory biomass, but the results are inconsistent. Understory biomass is negatively correlated with stand age^[99]^ due to weakening the light resource and increasing intraspecific competition. However, changes in understory biomass are inconsistent across different successional stages, and it generally increases in the early successional stages^[100]^. The current evidence is insufficient to understand the temporal dynamics of understory biomass.

The lack of practical and accurate assessment methods limits understory vegetation biomass assessment. The traditional destructive harvesting and oven-drying method is the most accurate method to measure understory biomass. However, it is labor-intensive and time-consuming, disturbs the soil environment, and hinders successive monitoring, especially for long-term experiments. The application of biomass estimation models is a non-destructive alternative in which biomass is estimated based on easily measured attributes of a plant or community. This method helps weaken the harm to vegetation and trace the long-term changes in stand biomass. Biomass allometric equations for understory vegetation mainly include species-specific and mixed-species models based on the number of species to be modeled^[101,102]^. The species-specific model is mostly individual- or species-level allometric model for specific dominant shrubs, which generally use relatively easy-to-obtain filed measurements of biometric data (e.g., crown diameter, percentage cover, height, stem basal diameter, or their combinations of two or three parameters), as a biomass predictor variable of shrub individuals^[102−104]^. This model is widely used in plantations with a good predictive capacity for dominant species^[102]^. However, this model is unsuitable for estimating the biomass of all species due to higher biodiversity in the understory layer, resulting in considerable uncertainty. Mixed-species model is generally established for a specific plant functional group (e.g., shrubs and herbs) using corresponding community structure parameters (e.g., percentage cover, average height, and average stem basal diameter)^[105,106]^. Jin & Bao^[105]^ fitted an optimal mixed-species model for the understory layer in a cypress plantation using the volume calculated as percentage cover multiplied by the average height. Although the accuracy may be partly lost due to great variations in morphology and structure of understory species (e.g., much-branched and tillering), the mixed-species model dramatically improves estimation efficiency and widen the range of applications. Presently, the mixed-species model is rarely used and needs more attention.

Understory vegetation, as a substantial nutrient pool, is an essential contributor to nutrient cycling due to its rapid turnover and the large proportion of high-quality (low C/N ratio) and easily decomposable litter^[107,108]^. Nutrient storage patterns are generally similar to biomass storage trends because understory biomass determines nutrient storage. However, plant nutrient content may affect the estimation accuracy of nutrient storage. A global synthesis showed that woody plants have higher C contents (averaged 48.0%) than herbaceous plants (averaged 43.1%); woody organ C contents range from 47.4 to 48.6%, while herbaceous organ C contents range from 42.4 to 44.7%^[109]^. Notably, this study, with small samples, did not identify the C contents of shrubs. Plant or community C storage may be overestimated using the widely employed C content of 50%, especially for understory vegetation. The ratio of the understory layer C storage to forest total C storage ranges from 2.2 to 18.8%, and the ratio for N ranges from 6.0 to 26.0% in different subalpine conifer forests^[110]^, indicating that the contribution of understory could not be neglected. Other nutrient contents (e.g., N, P, potassium (K), magnesium (Mg)) in the understory are higher than those in overstory trees, and herbaceous species have the highest amounts of nutrient contents^[3,20,111]^. Landuyt et al.^[112]^ found that N and P contents of understory vegetation in temperate deciduous forests averaged 2.7% and 2.7‰, respectively. These findings are mainly focused on specific understory dominant species or forest types, leading to significant uncertainties in nutrient storage assessment. Therefore, we suggest that the specific nutrient contents of various organs, life forms, and forest types across climatic regions should be considered in estimating nutrient storage. Although the understory layer C storage has been intensely studied at the stand or regional scales^[113]^, the generalization of other nutrient storage is less understood. Moreover, site-specific single-point studies may be inaccurate and incomplete against various environmental conditions. Synthesis analysis, such as the meta-analysis method, would be an optimal option in assessing the relative contribution of understory to forest nutrient storage in the context of climate change and land use.

## Carbon and nitrogen relationship of understory species

Plant species in the understory largely depend on maintaining a positive net C balance under shade conditions (i.e., Carbon Gain Hypothesis^[114]^). Leaf N is a determining factor of understory plant C assimilation, as N is a dominant component of the photosynthetic apparatus (carboxylation enzyme, Calvin proteins, and leaf chlorophyll)^[115]^. Generally, leaf N concentration in understory species is higher than in overstory tree species^[116]^, corresponding to the maximization of C gain under light limitation. Alternately, root N uptake is a C-cost process. More C would be allocated to the roots of understory plants when assimilate production is enhanced^[117]^. Therefore, understory plant C gain and N uptake are well coupled. However, global changes (e.g., greenhouse emissions, warming, drought, and N deposition) can modify the relationships between understory plant C gain and N uptake and consequently challenge biodiversity and the net primary productivity (NPP) of forest ecosystems^[118,119]^. Here, we reviewed the effects of main global changes on understory plants' C and N relationships.

Carbon dioxide (CO_2_) is one of the critical substrates of plant photosynthesis. The concentration of CO_2_ is relatively sufficient for understory plants due to the lowered maximum light-saturated photosynthetic rates under limited light conditions. In the context of climate change, elevated CO_2_ (e[CO_2_]) may increase the photosynthetic light use efficiency of understory plants to compensate for the restrained leaf C gain^[120]^. However, down-regulation of photosynthesis has also been observed in understory seedlings under e[CO_2_]^[121]^. Such inconsistency can be partially explained by the difference in N partitioning regimes among understory species grown in diverse environments^[122]^. Generally, photosynthesis and growth are temporarily stimulated by e[CO_2_] but attenuated in the long term because plants are unable to acquire sufficient N^[123]^. For optimizing N use efficiency under e[CO_2_] conditions, a significant fraction of leaf N is invested in the photosynthetic apparatus^[124]^. For example, the dwarf bamboo increased concentrations of both carbohydrate and N for e[CO_2_] acclimation, indicating the close relationships between C gain and N utilization in the understory^[125]^. The free-air CO_2_ enrichment (FACE) experiments have also provided direct evidence for the link between photosynthesis acclimation and N supply under e[CO_2_] concentrations^[124]^.

Global warming can affect plant survival and growth, community structure, ecosystem NPP, and consequently terrestrial C sequestration^[126]^. Significant thermophilization (warm-affinity) of understory plant communities has been documented, reflecting an increase in species adaptation ability to warmer conditions^[119]^. Macroclimate warming (of the free atmosphere) can affect microclimates (of the understory), and *vice versa*; the local microclimatic effects of the understory could regulate the impacts of macroclimate warming^[126]^. Warming generally stimulates enzymatic activities in plants (e.g., improved photosynthetic enzyme activity for C fixation and nitrate reductase for N uptake), allowing understory species to quickly approach the optimal temperature of photosynthesis as a consequence of improved growth and reproduction. Soil warming in the context of global warming also plays a vital role in soil nutrient availability, especially N nutrition^[127]^. An increase in soil temperature can promote microbial activity, further stimulating the decay rate of plant organic matter and N mineralization^[128]^. Thus, understory plant N availability can be enhanced and N limitation will be relieved under soil warming, leading to improved N uptake by root. Not only is root N absorption affected by global warming, but the leaf-level C assimilation can be altered by heterogeneous microclimates in the understory. Understory species growth is characterized by thermal limitation due to the reduced solar radiation by the canopy. A field study pointed out that high light supplements can boost understory plant responses to warming due to limited light availability and temperature under closed-canopy forests^[118]^. In addition, N fertilization combined with warming did cause strong community responses (i.e., biodiversity) of understory, although extra N inputs alone had only minor effects in N-saturated temperate forests^[118]^. It remains unclear how warming interplays with N addition affecting the understory plant C gain and N uptake in N-limited forests.

In the understory, plants are mostly characterized by shallow root systems, which makes them particularly susceptible to drought and nutrient-poor soils. Drought can challenge plant hydraulic conduction, decrease leaf photosynthetic capacity and production, and distinctly modifies plant C and N relationship^[129]^. Assimilates transported from the source (leaf) to the sink (root) are simultaneously stopped or reduced on account of drought-induced hydraulic failure^[130]^. Meanwhile, drought-induced xylem embolization commonly reduces water transportation from roots to aboveground parts, which further restrains N transportation in woody seedlings in the understory. Drought also reduces root nutrient uptake and nutrient mobilization in soil^[131]^ and inhibits N mineralization due to restrained microbial metabolic activities^[132]^. Besides, warmer air temperatures and increased vapor pressure deficit with the open canopy influence the habitat microclimate and resource availability where understory seedlings grow^[133]^, further intensifying the adverse effects of water and nutrient deficiencies. As a whole, drought can decouple the above- and below-ground C and N relations of understory seedlings, which may potentially regulate forest ecosystem production and nutrient cycling.

N deposition is one of the major issues of global change. An increase in N input can stimulate plant N uptake and contemporarily improve leaf C assimilation by enhancing soil N availability^[134]^. Meanwhile, additional nutrients also increase the capacity of understory plants to utilize the sporadic burst of high irradiance in sunflecks^[135]^. Extensive research has been conducted on understory and/or canopy N additions to simulate the effects of atmospheric N deposition on understory plants' C allocation and N uptake^[136−138]^. Due to specific differences in N sensitivities^[116]^, N deposition may even change the understory community structure through enhanced species' survival with high N tolerance and low light requirements. Huo et al.^[139]^ reported that the survival rate and the competitiveness of *Q. aliena* seedlings in the understory can be improved by increased soil N in the pine-oak mixed forest, promoting the succession of pine (*P. tabulaeformis*) forest to oak (*Q. aliena*) forest^[140]^. Moreover, increased growth and cover of canopy trees under N enrichment may reduce light availability for understory plants, suppressing leaf photosynthesis. For instance, Mao et al.^[137]^ found that understory plant growth might be more limited by light than N in tropical reforested ecosystems. Furthermore, N deposition in tropical regions may accelerate ecosystem P limitation, which can decline the photosynthetic performance of some understory species in N-rich tropical forests^[141]^. Thus, it is necessary to consider the combined effects of various nutrients in determining understory plant C gain and N uptake.

**Figure 4:**
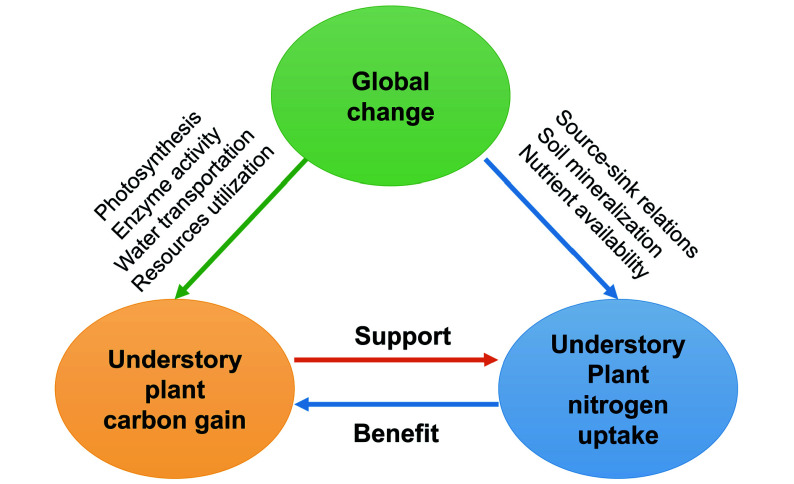
A framework of global change effects on carbon gain and nitrogen uptake by understory vegetation.

Currently, studies are still very scarce about understory plants' C and N relationships in responses to interactions among global change factors^[3]^. Here, we suggest that: i) global changes should be taken into account in synchronisation to reveal the underlying mechanisms of understory plant C gain and N uptake responses; ii) integrated investigation of understory functional responses (C and nutrients utilization and cycling) will be crucial to predicting functional changes in the understory comprehensively. Developing a conceptual framework synthesizing the possible effects of multiple global changes will benefit our understanding of the driving mechanisms mediating forest ecosystem functioning (Fig. 4).

## Functional traits of understory species

**Figure 5:**
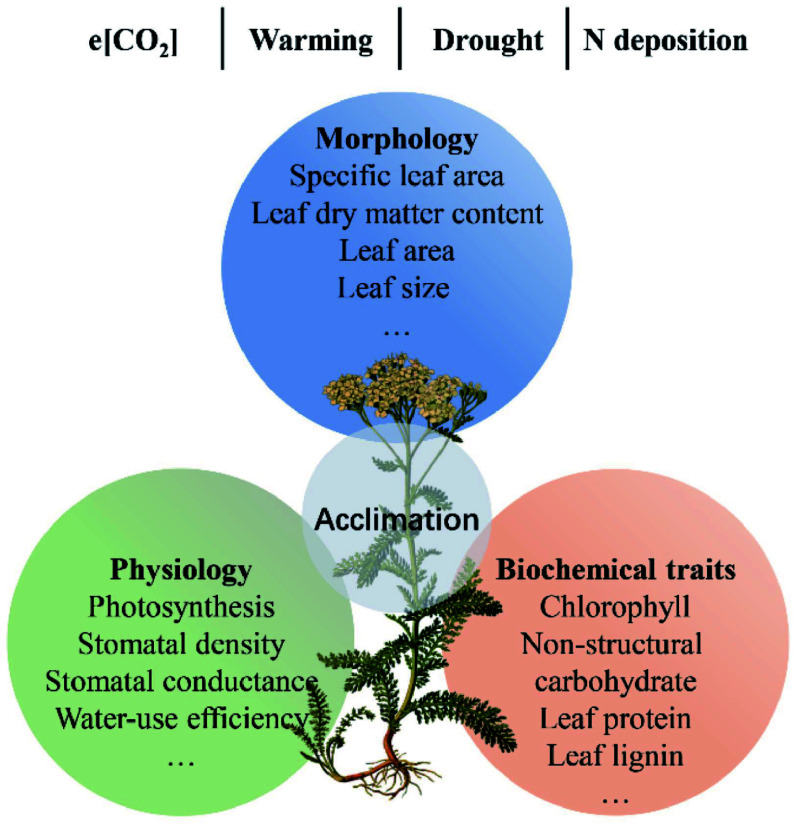
The overview on the responses of understory plant function traits to environmental changes in forest ecosystems.

Understory vegetation is susceptible to environmental changes and can alter functional traits to improve its adaptability, e.g., enhancing defense and/or resource acquisition^[136,142,143]^. A classic example is the specific leaf area (SLA) of understory plants, which can increase with increasing nutrient availability to enhance light acquisition^[143,144]^. Given the vital role of understory plants and their complex relations with changing environments, we reviewed recent advances in functional traits of understory species and their acclimation to environmental changes. We focus on the impacts of e[CO_2_], global warming, drought, and increased N deposition on leaf morphology, physiology, and biochemical traits of understory species (Fig. 5).

e[CO_2_] is conducive to plant growth due to the critical role of CO_2_ in photosynthesis (i.e., fertilization effect). Thus, e[CO_2_] can enhance the net photosynthetic rate but decrease total chlorophyll concentrations in leaves^[145]^. Consequently, e[CO_2_] could increase leaf area and decrease SLA due to C accumulation^[146]^. Numerous studies have found that e[CO_2_] can affect C allocation and distribution, altering the concentrations of chemical compounds^[125,146]^. For example, carbohydrate concentrations, such as sucrose, sugar, starch, and non-structural carbohydrate (NSC), have been increased by long-term e[CO_2_]^[125,146]^. In addition, e[CO_2_] can also affect the process of C-water exchange. For instance, e[CO_2_] can decrease leaf stomatal conductance, resulting in low water loss and high plant WUE^[125]^.

The physiology and ecology of forest understory species are often highly sensitive to changes in temperature^[147]^. However, the effects of warming on understory vegetation are still unclear^[147,148]^. On the one hand, previous studies have found that experimental warming can promote individual height and productivity of understory plants^[148]^. Since plants under high temperatures had an early onset of leaf senescence, on the other hand, some studies also found that warming can decrease photosynthesis, stomatal conductance, and total biomass^[147]^. For leaf traits, enhanced temperature decreases leaf dry matter content, nutrient and pigment concentrations, SLA, and stomatal parameters^[149]^. In addition, plant species have remarkable differences with contrasting life forms in response to increased temperature^[148]^. For example, warming affects understory herbs than woody species^[150]^.

The frequency and intensity of drought will increase in the future, which can result in plants undergoing several changes. In general, drought significantly affects plant water cycles^[151]^. Leaf water potential is negatively affected, while leaf C isotopic composition (or intrinsic WUE) is positively affected by the drought^[151]^. Moreover, drought stress can decrease leaf size, stomatal number, and leaf surface, and thicken cell walls, profoundly inhibiting the leaf biomass, plant height, and aboveground biomass of understory plants^[152]^. The accumulated evidence indicates that the response of understory plant functional traits to drought depends on the climate zone and plant functional groups. For instance, leaf photosynthesis is generally insensitive to drought in temperate forests but sensitive in rainforests, leading to the depletion of leaf starch in shrubs of understory plants^[56]^. Drought decreases the SLA of grasses but not that of forbs in temperate systems, which might be associated with a phenotypic adjustment that enhances WUE under water stress^[153]^. In sub-Mediterranean systems, however, grasses significantly increase SLA under drought conditions, which might be related to their strategy to allocate resources to belowground parts^[153]^.

N deposition has dramatically increased since the 1980s, exerting huge impacts on functional traits and acclimation of understory plants^[136,154,155]^. Previous studies have shown that variations in SLA, leaf dry matter content, leaf nutrient concentrations, and chemical compounds are closely related to N deposition^[136]^. First, increased N deposition can reduce the light availability of understory plants due to an increase in canopy coverage; thus, understory species could enhance SLA and chlorophyll to increase light capture ability^[144]^. Second, increased N deposition can increase leaf N but decrease P concentrations and the contents of nutrient cations (e.g., K^+^, Ca^2+^, and Mg^2+^), which might lead to a risk of N-meditated nutrient imbalance^[156]^. Third, increased N deposition can reduce lignin while increasing concentrations of leaf organic acids, protein, and NSC of six understory species^[136]^. Additionally, Mao et al.^[156]^ found that N addition increased the soluble protein and/or free amino acids in understory plants but lowered photosynthesis capability since nutrient imbalance. Furthermore, the response of understory plants to N deposition also differs among plant functional groups. It has been shown that medium-light and shade-tolerant species have a minor physiological response to N addition, but shade-intolerant species (i.e., *Alchomea trewioides*) are sensitive^[156]^.

Although the responses of understory plants to global changes have been widely studied over the past century, most studies focused on a single factor. It might not be sufficient to uncover the impacts of global changes on understory plants since those changes in a given habitat always coincide in nature^[157]^. Their effects can be antagonistic or synergistic. Therefore, there are some crucial questions for future attention. First, more work is necessary to explore how multiple environmental changes affect understory plants' functional responses and acclimation. In this aspect, both earth system models and multiple-factor experiments may be good tools. Second, untangling causality between understory and canopy plants is also urgent. The correlations between the understory and canopy communities remain poorly understood, resulting in a considerable gap in revealing how environmental changes affect understory plants and forest ecosystem functioning. Together, it is necessary to further reveal the responses of plant functional traits and their contributions to understory plant defense and growth under multiple global changes.

## Litter decomposition of understory vegetation

Litter decomposition is a primary biological process which is the key to understanding the global C cycle^[158,159]^. Understory vegetation only accounts for a small fraction of forest biomass but contributes significantly to litter biomass accumulation and nutrient recycling^[160−162]^. According to the well-known triangular model, litter quality (e.g., lignin and C/N ratio), climatic conditions (e.g., temperature and moisture), and decomposers (e.g., soil fauna and microorganisms) all drive global litter decomposition^[163,164]^. In this paradigm, litter decomposition of understory vegetation may follow a distinct trajectory from canopy trees, and its decomposition rate varies with environmental conditions, ecosystems, and vegetation types^[165]^. Global change has further increased the uncertainty of litter decomposition of understory vegetation^[166]^. However, the litter decomposition of understory vegetation has been largely ignored in previous studies of forest litter decomposition. Here, we reviewed 'trees-shrubs-herbs' decomposition in a mixed litter composite, highlighted the unique microhabitats and decomposers upon litter decomposition associated with understory vegetation, and projected the future study's focus on improving our understanding of the C cycle of forest ecosystems.

Compared with the decomposition of solely tree litter, taking understory vegetation litter into consideration pioneers serial studies of 'trees-shrubs-herbs' mixed litter. According to the theory of the 'resource complementarity effect', understory vegetation can substantially promote the decomposition of tree litter^[167−169]^. For example, Fujii et al.^[168]^ reported that tree species diversity did not significantly change the litter decomposition rate of trees in northern Japan, but there was an apparent synergistic effect when mixed with understory herb litter. A similar case was recently found in southwestern China, where understory litter significantly promoted mixed litter decomposition with trees^[167]^. Inconsistent results, however, were reported by Roeder et al.^[170]^, who showed that vine litter did not affect the decomposition of mixed litter with tree plant species. Decomposition of the mixed litter, particularly for a mixture of understory and tree litter (i.e., a highlight on 'trees-shrubs-herbs' decomposition), is thus pending further exploration towards a deep understanding of the mechanisms that underlie their patterns. These mechanisms include but are not limited to: i) Nutrient transfer and resource complementarity. Nutrients transfer from nutrient-rich understory vegetation litter to nutrient-poor tree litter and accelerate the decomposition process of the mixture^[169,170]^; ii) Changes in the microclimate. The mixed litter usually has a higher water-holding capacity, which benefits the decomposition^[171,172]^; iii) Feedback regulation of food resources by decomposers. The higher the diversity of litter species is, the richer the decomposition substrates it provides for microorganisms and soil fauna, resulting in a faster biodegradation process^[167,173,174]^; iv) The presence of specific compounds. Secondary metabolites in one litter (e.g., polyphenols) can promote or limit the activity of decomposers and alter the decomposition^[175,176]^.

**Figure 6:**
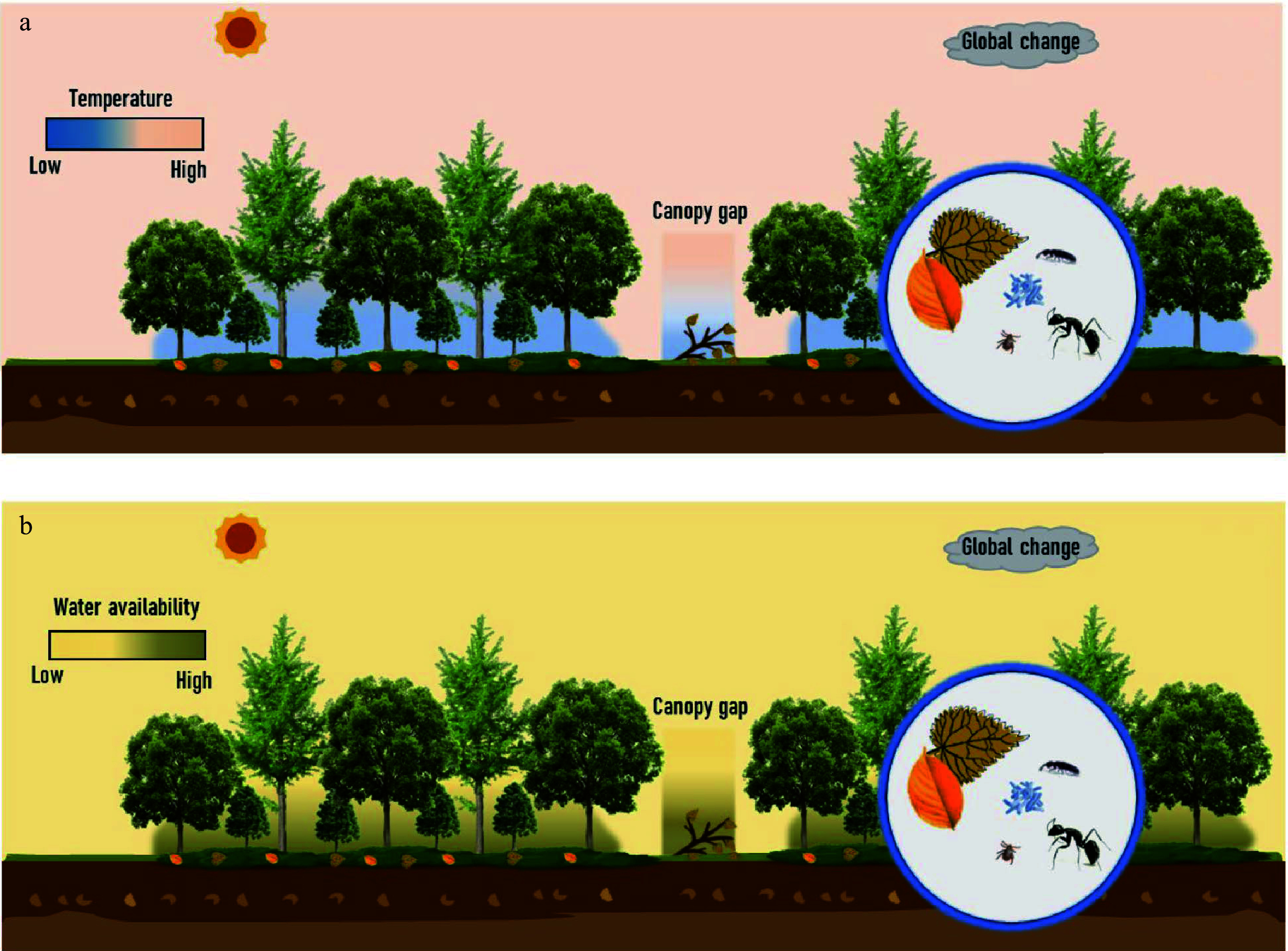
Litter decomposition of understory vegetation and potentially drivers. The potential drivers include litter quality in a 'trees-shrubs-herbs' mixed litter, microclimates (e.g., temperature, moisture contents, and light), and decomposers (e.g., microorganisms and soil fauna) in the context of global changes, all in a close relatedness to understory vegetation. (a) Difference in temperature and the other potential drivers among the overstory, understory, and canopy gap. (b) Difference in water availability and the other potential drivers among the overstory, understory, and canopy gap. Two panels highlight the unique habitats of the forest understory.

Beyond the direct effect of understory vegetation litter mixed with those from trees, understory vegetation provides unique habitats, including microclimates (e.g., temperature, moisture contents, and light) and ecological niches (e.g., shading and physical interception)^[177−179]^, thus indirectly impacting on the processes of understory vegetation litter decomposition (Fig. 6). On the one hand, shading by understory vegetation can increase the understory water-holding capacity and keep the understory water relatively stable, benefiting the decomposition of litter in water-limited forests (e.g., semi-arid and karst forests)^[180]^. On the other hand, photodegradation driven by solar radiation has been identified as another important driver controlling litter decay and C cycling across various ecosystems^[181]^. Although solar irradiance is relatively low in the forest understory, there is increasing evidence that photodegradation promotes litter decomposition in mesic forest ecosystems, e.g., tropical^[182]^, subtropical^[183]^, temperate, and boreal forests^[184−186]^. For instance, a recent study in a temperate forest showed that photodegradation significantly increased litter mass loss by 120%^[186]^. Photodegradation may be pronounced for a considerable part of the standing woody and leaf litter that remained in no contact with grounds due to the physical interception of understory vegetation. Moreover, canopy (understory *vs* gaps) and season (open *vs* closed canopy phenology) can greatly modulate the effect of solar radiation on nutrient dynamics during litter decay^[187]^. However, such an unexpected impact of solar radiation on the C cycle in the understory is generally ignored in the previous estimation of the terrestrial C budget. The interactive effect of understory microclimate and ecological niches, especially interactions between understory water holding capacity and photodegradation, on understory litter decomposition needs particular attention.

Litter, microorganisms, and soil fauna make up the basic understory detritus food web, where top-down and bottom-up control litter decomposition and their decomposers during decomposition^[169,188,189]^. Litter diversity can affect decomposer diversity from the bottom to the top *via* the food web^[190]^, which provides vital feedback on litter decomposition. Conversely, soil fauna and microbial diversity can influence litter decomposition from top to bottom^[189]^. These theoretical perspectives, however, have seldom been considered in the field of understory litter decomposition, calling for studies focusing on coupled litter diversity and decomposer diversity with combined molecular techniques (e.g., high-throughput sequencing and eDNA) and isotopic labeling techniques (e.g., ^13^C and ^15^N). In addition, leaching and fungal mycelia are considered two major pathways responsible for transferring nutrients among mixed litters^[175,191,192]^, but how these pathways work together to drive the nutrients from one vegetation litter to another is unclear. Indeed, the transfers of C and other nutrients among mixed litter differ depending on litter materials and local niches^[173,175]^. The transfers of various C components are also not precisely known^[167,193]^, which impedes the accurate prediction of nutrient transfer among the mixture. Further study should innovate the methodology issues concerning the additive and non-additive effects behind mixed litter decomposition.

Global changes can profoundly affect understory litter decomposition directly or indirectly by altering the understory microclimate, vegetation functional structure, and decomposer activities^[166,194−198]^. For example, according to a meta-study, warming increased the rate of litter decomposition by 4.2% on a global scale^[197]^. The influence of e[CO_2_] on litter decomposition is relatively inconspicuous compared to that of temperature rise, but e[CO_2_] can remarkably change C and other chemicals in fresh leaves, thus mediating the trajectory of litter decomposition through modifying litter quality^[199]^. Moreover, N availability drives both plant productivity and litter recycling, and the responses of understory litter decomposition to N deposition are closely related to its levels. Low levels of N input commonly tended to promote litter decomposition, but high levels tended to produce inhibition effects^[200]^.

Nevertheless, global changes also can synchronize to produce strong interaction effects upon decomposition processes. For example, photodegradation is a contributing driver of litter decomposition across biomes^[181]^, and the relevant role of photodegradation may explain why C cycling is underestimated by the empirical models in terrestrial ecosystems. It is projected that the exposure of plant organic matter to solar radiation will be significantly modified by interactions of stratospheric ozone, climate (e.g., cloud cover), and land use (e.g., deforestation) at the global and regional scale^[201]^, and by forest canopy structure and phenology, and understory vegetation at the stand scale^[187]^. A better understanding of how understory sunlight controls C and nutrient dynamics in forest ecosystems is essential to accurately assess the response of global biogeochemical cycles to climate changes. Therefore, how multiple global change factors jointly drive understory litter's decomposition and nutrient release demands more attention.

## Understory vegetation and overstory tree interactions

The interaction between under- and over-story plays a critical role in driving the composition, structure, and function of forest ecosystems^[1]^, which is central to understanding the mechanisms of species coexistence, biodiversity maintenance, and forest management^[202,203]^. Their interaction is generally sustained under competitive conditions for resource availability, such as light, nutrients, and water^[204,205]^, encompassing a range of negative and positive relationships by forming adaptive gradients from inhibition to tolerance and facilitation^[7,206]^. These relations can also change dramatically in the face of environmental variability^[12,207,208]^. This motivates the urgency of exploring the potential driving mechanisms of reciprocal under- and over-story interactions^[9]^, especially since only a few studies have focused on the link of understory vegetation to overstory trees^[202,209]^. Here, we reviewed recent advances in the interactions among multiple vegetation layers managed mainly by understory removal (i.e., bottom-up control) and tree thinning/pruning (i.e., top-down control) because both are widely applied strategies in current forest management.

Photosynthetic physiological responses of overstory to understory management are essential for tree growth, regeneration, and productivity^[210,211]^. A few studies showed that understory vegetation removal elicited large increases in photosynthetic characteristics, such as net CO_2_ assimilation rate, stomatal conductance, transpiration rate, as well as N, P, and other nutrients use efficiency in coniferous (e.g., *P. densiflora* and *Cunninghamia lanceolata*) and broadleaved (e.g., *Eucalyptus globulus* and *B. ermanii*) trees, but a decrease in WUE^[7,212,213]^. These changes were pronounced in the upper leaves, whereas the photosynthetic performance of the leaves from the middle to lower canopy was not significantly affected^[214]^. However, others have reported no effect on photosynthetic capacity in the absence of understory vegetation^[215,216]^. In addition, C balance (gain and utilization), commonly characterized by NSC dynamics, is another critical factor affecting tree growth^[217,218]^. Understory removal does not affect NSC concentration and their fractions (soluble sugar and starch) in both the above- and below-ground organs/tissues (e.g., needle, xylem, inner-bark, and fine root) of coniferous trees^[216,219]^. However, it is in contrast to a positive increase in tree NSC pools that differ between the dry season and the rainy season in a mid-story removal experiment^[210]^. To a large extent, photosynthetic C processes are associated with changes in leaf functional traits of overstory trees^[203]^, particularly SLA, which responds more strongly to understory removal at the leaf level^[220, 221]^.

The dynamic demand of understory vegetation for environmental resources often controls the growth and natural regeneration of overstory trees. Understory vegetation removal can improve relative growth rates, e.g., annual increments of trunk diameter, height, basal area, and stand volume for desirable tree species, including their seedlings, due to enhanced SLA and foliar photosynthetic physiology^[7,13,210,221−224]^. A meta-analysis showed that the magnitude of the increase in tree growth mediated by understory removal depended on the tree's development stage and treatment applied age^[225]^. This status greatly increases the aboveground biomass and productivity^[226−228]^, despite a negative relationship with inherent site fertility. Subsequently, understory removal has positive effects on the natural regeneration of overstory trees (i.e., emergence, survival, reproduction, and growth)^[220,229]^, in which early-successional tree species are more strongly affected by removal than mid- or late-successional species (see review in De Lombaerde et al.^[14]^). However, the growth rate of overstory trees does not benefit from understory vegetation continuously absent at a long-term stage^[215,224]^. It even decelerates at the stand level^[6,216]^, irrespective of photosynthetic performance. The increment in growth rates tends to disappear in small-diameter trees, possibly due to a strong suppression from larger-diameter trees at a specific stand density and age after removal^[216,224,230]^. Consequently, the effect of understory on the growth and regeneration of overstory trees is likely to be moderate, nil, or severe.

The effects of overstory trees on understory strata have been fully identified in the last two decades^[5,9]^. Tree thinning/canopy trimming alone or in combination with other abiotic factors, such as wildfire, drought, and grazing, results in various ecophysiological responses in understory vegetation through heterogeneous microhabitats^[10,231]^. In general, previous studies have mainly focused on the impacts of overstory management on understory vegetation and fractional cover growth rate^[211]^, composition^[208]^, diversity^[232]^, photosynthetic characteristics^[233]^, biomass allocation^[107]^, and regeneration^[234]^. However, changes in overstory trees may induce a positive to neutral to a negative effect on understory vegetation characteristics at the plot scale or stand scale, as understory species vary from bryophytes to grasses to shrubs in varying plantations^[1,11]^. Moreover, existing studies were conducted either in natural stands and mature plantations or in very young plantations. They completely neglected long-term monitoring of overstory on understory vegetation from the seedling stage through tree canopy closure^[235]^, which is an essential process in determining stand development and ecological function.

**Figure 7:**
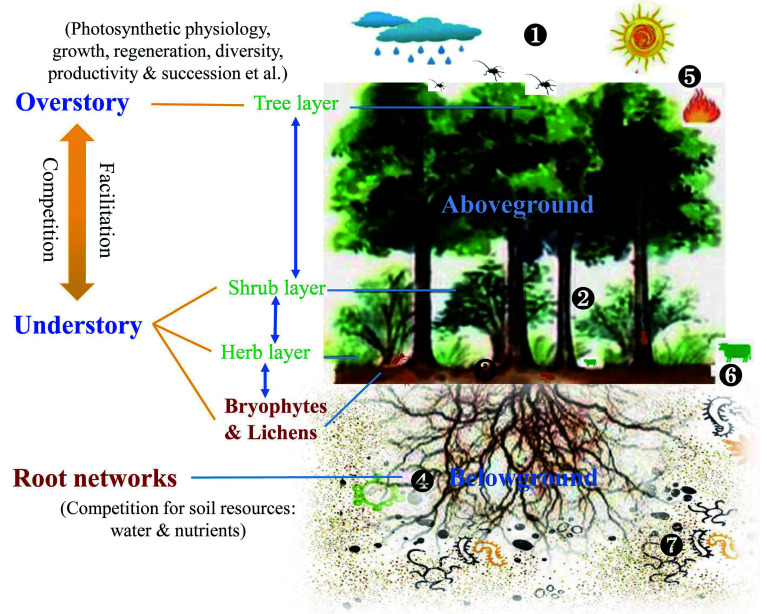
Interactions between understory vegetation and overstory trees. Key regulation processes are shown: 1) Light and rainfall interceptions by overstory change the understory microclimate; 2) Composition and diversity of understory vegetation influence preconditions for seed germination and regeneration of overstory; 3) Under- and over-story interactions affect ground surface arthropods, surface runoff and litter decomposition; 4) Root interactions between under- and over-story, including mycorrhizal network, root exudate and allelopathic effect, alter belowground water and nutrient cycling as well as information transformation; 5) Abiotic and biotic factors such as drought, fire, and insect pests, directly influence the dynamics of the whole forest community; 6) Large mammalian herbivores and some arthropods also directly feed on understory vegetation; 7) Soil micro-food webs feed on roots (i.e., root-feeding nematodes) and consume related residues, thus resulting in changes in soil environments. These above- and below-ground processes can shape under- and over-story interactions, thereby influencing their photosynthetic physiology, growth, regeneration, productivity, and the like. However, bryophytes and lichens are always overlooked, and the root system has also received little attention.

Overall, the differential effect of understory management on overstory trees, or *vice versa*, may be controlled by multiple factors (Fig. 7), such as species types (e.g., coniferous *vs* broadleaved tree), stand age (e.g., mature *vs* young stand), planting density (e.g., low *vs* high density), site attributes (e.g., mesic *vs* hydric site), and management timing and duration^[202,236]^. Three primary processes driving under- and over-story interactions were proposed: i) Overstory effect: canopy openness would affect light and rainfall interceptions, leading to differences in the light quality and water availability utilized by understory species; ii) Understory effect: the dense understory vegetation can shape surface microenvironments (e.g., temperature and moisture) and several critical ecological processes, such as litter decomposition and nutrient mineralization, thus influencing soil resources uptake of trees; iii) Belowground effect: complex roots interactions including their mycorrhizal networks extend the soil volume explored by over- and understory, transfer resource and information^[8,9]^. Furthermore, the allelopathy of plant-produced phytochemicals induced by plant invasion or agroforestry also alters under- and over-story interactions through direct interspecific competition for soil resources or indirect effects on the nutrient cycling of forest ecosystems^[213,223]^. It is worth noting that these effects tend to co-occur, and their importance should be distinguished in a comprehensive study.

Under- and over-story interactions, a persistent topic in forest ecology, are complex and asymmetric. However, there are still significant gaps in our knowledge, which need to be explored on a large scale from the community ecophysiology to belowground networks in an integrated study. Thus, we answer the following questions to better understand the under- and over-story relationships under different management practices, especially in climate change. First, plant ecophysiological processes are major determinants of growth and development, influencing community dynamics and productivity. Few studies have specifically assessed community ecophysiology at the canopy level through different tissues/organs, including fine and coarse roots, in response to thinning or understory removal. Moreover, the contributions of the bryophyte and lichen layers are completely ignored. Therefore, it is necessary to strengthen the study of hierarchical interactions among multiple vegetation layers, which would provide insight into C balance and nutrient cycling in forest ecosystems. Second, in addition to competition for light and water, the cross-talk between neighboring roots of under- and over-story plants, especially involving mycorrhizae, is crucial for regulating soil structure and resources and alleviating abiotic stress in forest understory communities. However, it remains unclear how mycorrhizal networks, root exudates, and microbiota shape the belowground process and intervene in under- and over-story interactions. Finally, due to the uncertainty in under- and over-story interactions, long-term studies towards joint management or in combination with climate change (e.g., warming, drought, and N deposition) need to be further explored, consequently gaining a finer understanding of the spatiotemporal variability of the interactions. An isotope tracing approach is strongly recommended to shorten the experimental period and improve our knowledge of interaction processes.

## Understory vegetation effects on soil chemistry

**Figure 8:**
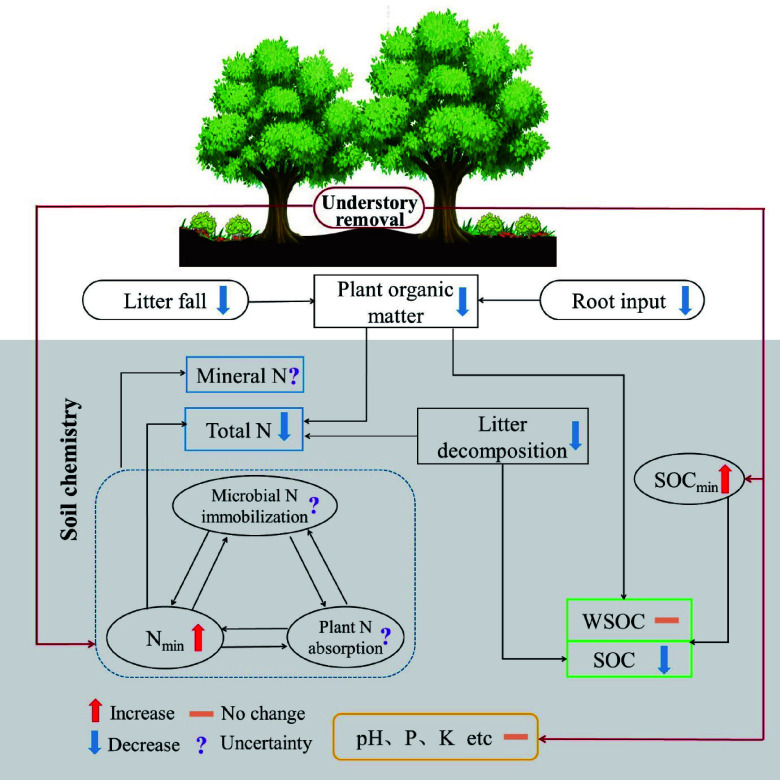
Conceptual diagram illustrating the effects of understory removal on soil chemistry. The symbol to the right of the parameter represents the possible response of the parameter after understory removal based on literatures. The upward and downward arrows represent the promotion and inhibition effects, respectively; the short horizontal line indicates that the influence is not significant, and the question mark represents the uncertain effect. SOC_min_, soil organic carbon mineralization rate; N_min_, soil net nitrogen mineralization rate; WSOC, water-soluble organic carbon; N, nitrogen; P, phosphorus; K, potassium.

Understory vegetation is essential in promoting forest soil nutrient cycling and regulating soil chemical processes by directly and indirectly changing soil nutritional status^[226,229,237,238]^. The root exudates and litter of the understory strongly affect soil nutrient content and availability^[239]^. Moreover, understory vegetation can indirectly influence soil chemical properties due to its effects on the ground environment, such as reducing solar radiation, lowering soil temperature, and increasing soil water content^[240]^. Research on the understory function is primarily conducted in plantation ecosystems^[241−243]^ because it is generally removed to reduce its resource competition with target tree species in long-term forestry practices. Increasing biomass and diversity of understory vegetation are generally regarded as important ways to improve soil fertility in plantation forests^[244,245]^. Here, we reviewed recent advances in the influence of understory vegetation on soil chemistry, which is mainly reflected in soil pH and the concentration and dynamics of SOC, N, P, K, and other vital elements (Fig. 8)^[241,246]^. It would provide the scientific basis for understanding forest management and ecosystem functioning responding to climate change and land use.

Soil pH is one of the most pivotal soil chemical properties, affecting soil chemical and biochemical processes. Most studies have shown that understory removal in plantations had no significant effect on soil pH^[240,245,247]^. However, Fu et al.^[246]^ found that increased understory vegetation diversity elevated soil pH after 27 years of reforestation. Similarly, the invasion of some exotic understory plants in deciduous forests increased soil pH^[248]^. Additionally, Zhao et al.^[245]^ found that unless removing all aboveground vegetation, the removal of only understory vegetation did not change soil pH. These results suggest that understory vegetation may mildly regulate soil pH, but short-term understory removal cannot affect soil pH due to its smaller biomass.

Understory vegetation significantly drives soil C and N dynamics^[249,250]^. In general, understory vegetation contributes to soil C and N conservation. SOC would decrease following the removal of understory vegetation^[229]^, owing to a decrease in substrate input from roots and litter^[251]^, an increase in SOC decomposition^[241]^, and loss by surface runoff^[240]^. A decrease in SOC after understory removal is also related to a decrease in litter decomposition because understory removal decreases soil fungal abundance and increases the C limitation of microbial communities^[239]^. Although understory removal alters the quantity and quality of organic matter that inputs into the soil, the responses of SOC components to vegetation removal under the forest vary. Understory removal reduces soil particulate organic C and mineral-associated organic C concentrations in Chinese fir forests^[241]^. At the same time, the presence of ferns decreases the lignin concentration and increases the ratio of syring-based to vanilla-based lignin^[252]^. Meanwhile, understory removal decreases SOC but does not affect water-soluble organic C (WSOC) and microbial biomass C^[253]^. In addition, understory removal can also modify soil microenvironments (e.g., an increase in soil temperature), indirectly increasing organic matter mineralization and WSOC^[242,254]^. These suggest that understory vegetation should be preserved to maintain soil quality in forests, especially plantations, based on its role in forest C sequestration.

The effects of understory vegetation on soil total N (TN) and N availability are still debated. Some studies have shown that understory removal has a minor effect on soil TN, ammonium N (${\text{NH}}_4^+ $-N), and nitrate N (${\text{NO}}_3^- $-N)^[247,254,255]^. However, the significant response of mineral N and TN to understory removal is often detected, although the conclusions are inconsistent. For instance, understory removal increased ${\text{NH}}_4^+ $-N^[241]^, ${\text{NO}}_3^- $-N^[244]^, and mineral N^[241]^, while other study found the opposite results^[253]^. This inconsistency suggests that the understory vegetation effect on N availability appears to be comprehensively regulated by N mineralization, tree competition, and microbial immobilization. Previous studies reported that soil net N mineralization and nitrification rates increased after understory removal^[256−258]^ because of the reduction in microbial immobilization. Microbial growth is accompanied by N fixation, while understory removal reduces soil microbial biomass, decreasing in microbial N immobilization and increasing in net N mineralization^[259]^. In addition, the net soil nitrification rate may be limited by NH_4_^+^-N availability due to the competition from understory vegetation and nitrifying bacteria. After understory removal, a decreased plant N uptake may lead to a higher nitrification rate due to more N substrates for nitrifying bacteria^[241]^. However, reduced N mineralization and nitrification rates in subtropical forests after understory removal have also been detected because SOM is a source for heterotrophic microorganisms^[260]^. It may be interpreted that reduced SOM inhibits microbial N mineralization and nitrification processes^[240]^. Therefore, the mechanism of how understory affects soil N availability and mineralization remains to be further investigated.

Understory vegetation seems to have little effect on the other major elements in the soil except for C and N. Soil P is supplied by parent material, litter, and fertilizer input. Understory removal seems to have no significant effect on soil total and available P in plantation forests^[243,253,261]^, although it may affect a certain P fraction, e.g., the occluded P^[243]^. Like P, available K also appears less susceptible to understory removal^[240]^. Understory vegetation has a significant impact on soil C and N dynamics. At the same time, its effects on pH and other elements are limited due to the small proportion of understory biomass relative to trees. Additionally, understory vegetation may affect other soil chemical parameters, such as the cation exchange capacity and redox potential of soil, but relevant research has not paid enough attention.

Studies on the effects of understory vegetation on soil chemical properties have received widespread attention in plantation and natural forest ecosystems^[194,211]^. However, natural forests have high biodiversity, strong resistance to external disturbances, and more complex soil ecological processes than plantations. Whether the understory vegetation effect on soil chemistry is consistent in plantations and natural forests is worth investigating. Second, previous studies have mainly focused on extreme cases, that is, the impact of complete removal and retention of understory vegetation on soil ecological processes^[226,249,258]^. However, there are many intermediate degrees between the presence or absence of understory vegetation, such as the impact of understory biomass amount and biodiversity complexity on ecological processes^[246]^. Recently, several studies have established the relationship between understory biodiversity and soil properties, but there are relatively few studies on the regulatory mechanism of this relationship.

Furthermore, although we generally understand the understory role in soil chemistry, there is not enough mechanistic research on these results. For example, it is not fully understood how the quantity and chemical composition of litter, root exudates, and SOC, as well as the related microbial communities, are involved in C and N dynamics. In recent years, new technologies and methods, such as stable isotope technology^[252]^, microbial high-throughput analysis^[255]^, and organic matter grouping^[241,243]^, may provide new paths for the mechanism exploration in forest understory vegetation studies.

## Understory vegetation effects on soil microbial communities

Understory vegetation is closely associated with belowground ecological processes. Soil organisms are major drivers of many soil biogeochemical processes, including organic matter decomposition^[262]^, N mineralization, and SOC formation, playing an important role in maintaining and driving forest ecosystem function and productivity^[263]^. Forest understory vegetation with higher species richness can provide more complex and diverse living spaces and environments for soil organisms^[264]^. Its variation can directly or indirectly regulate soil organism communities and structure by changing the soil environment or supplying nutrient substrates from plant litter and root exudates^[265,266]^. Therefore, studying the interaction and feedback between understory vegetation and soil organisms is conducive to an in-depth understanding of ecosystem diversity, soil function, and forest ecosystem stability, especially for soil microorganisms, due to their importance and the greater attention they have received. Understory removal is an essential ecological component due to its modification of plant belowground C allocation and N supply as critical determinants of microbial community composition^[267]^. Here, we reviewed the coupled relationships between understory vegetation and soil microorganisms and the impacts of understory removal on soil microorganism dynamics.

The interaction between soil microorganisms and understory vegetation has received extensive attention under the driving forces of climate change and human activities. Most of studies indicate that the effect of understory vegetation on the soil microbial community depends on its composition. In general, understory removal decreases fungal phospholipid fatty acids (PLFAs) and fungal: bacterial ratio while having a minor effect on soil bacterial PLFAs in subtropical Chinese fir plantations^[268]^. Similar findings were obtained in subtropical *Eucalyptus* plantations^[264,269]^ and *Acacia mangium* plantations^[242]^. It might be related to the different nutrient requirements of soil bacteria and fungi^[270]^. However, in tropical *Eucalyptus* plantations, understory removal significantly increased bacterial PLFAs^[267,271]^. It suggests that the effect of understory vegetation on soil microorganisms varies according to climate type. However, previous studies have concentrated on subtropical and temperate low-altitude and tropical areas, while few are in boreal and high-altitude areas. In addition, the treatment duration of understory vegetation removal is another important factor. For instance, the biomass of soil arbuscular mycorrhizal fungi decreased after removing plant functional groups for five months in alpine shrub ecosystems, although this effect disappeared after 17 months^[271]^. It suggests that the understory effect on the soil microbial community is highly complex^[272]^. In addition, most of the previous studies are conducted at one or two sites, and the experimental designs are mutually independent; there is a lack of a general conclusions on the role of understory vegetation on influencing soil microbial communities on a large scale.

Furthermore, the mechanism of understory vegetation influencing soil microorganisms has been becoming a research hotspot. An increasing amount of studies have evidenced that changes in understory vegetation can directly affect soil microbial community *via* changing plant-microbe associations, quality and quantity of root exudates, and litter input, or indirectly affect the diversity and composition of soil microbial community through changing soil physical and chemical characteristics, such as soil moisture content, pH value, and nutrient availability (Fig. 7)^[273−275]^. However, the mechanisms by which understory vegetation alters microbial communities and influences microbial functions in forest ecosystems remain largely elusive. It would limit our understanding of how understory vegetation composition controls soil nutrient cycling by modifying soil microbial activities in a forest ecosystem. In addition, these findings are derived from some outdoor simulation control experiments, and whether these results can be applied to forestry management measures such as understory clearing needs further exploration.

As a whole, the mechanisms of forest understory vegetation driving soil microbial communities awaits further exploration. First, future research should focus on how the soil microbial community responds to changes in forest understory on a large scale, which could contribute to a better understanding of the impact of understory vegetation on the ecosystem function. Second, it is critical to quantify the drive pathways and mechanisms of understory vegetation on the soil microbial community to better understand the mechanistic links between belowground processes and aboveground productivity. In addition, the relative contribution of understory vegetation to soil bacterial and fungal communities should be quantitatively determined. Finally, community assembly processes are intrinsically associated with ecosystem functions owing to their essential roles in shaping the microbial community structure. The knowledge of how understory vegetation affects the soil microbial community assembly, thereby generating important feedback on ecosystem productivity, remains to be explored.

## Understory vegetation effects on soil and water conservation

**Figure 9:**
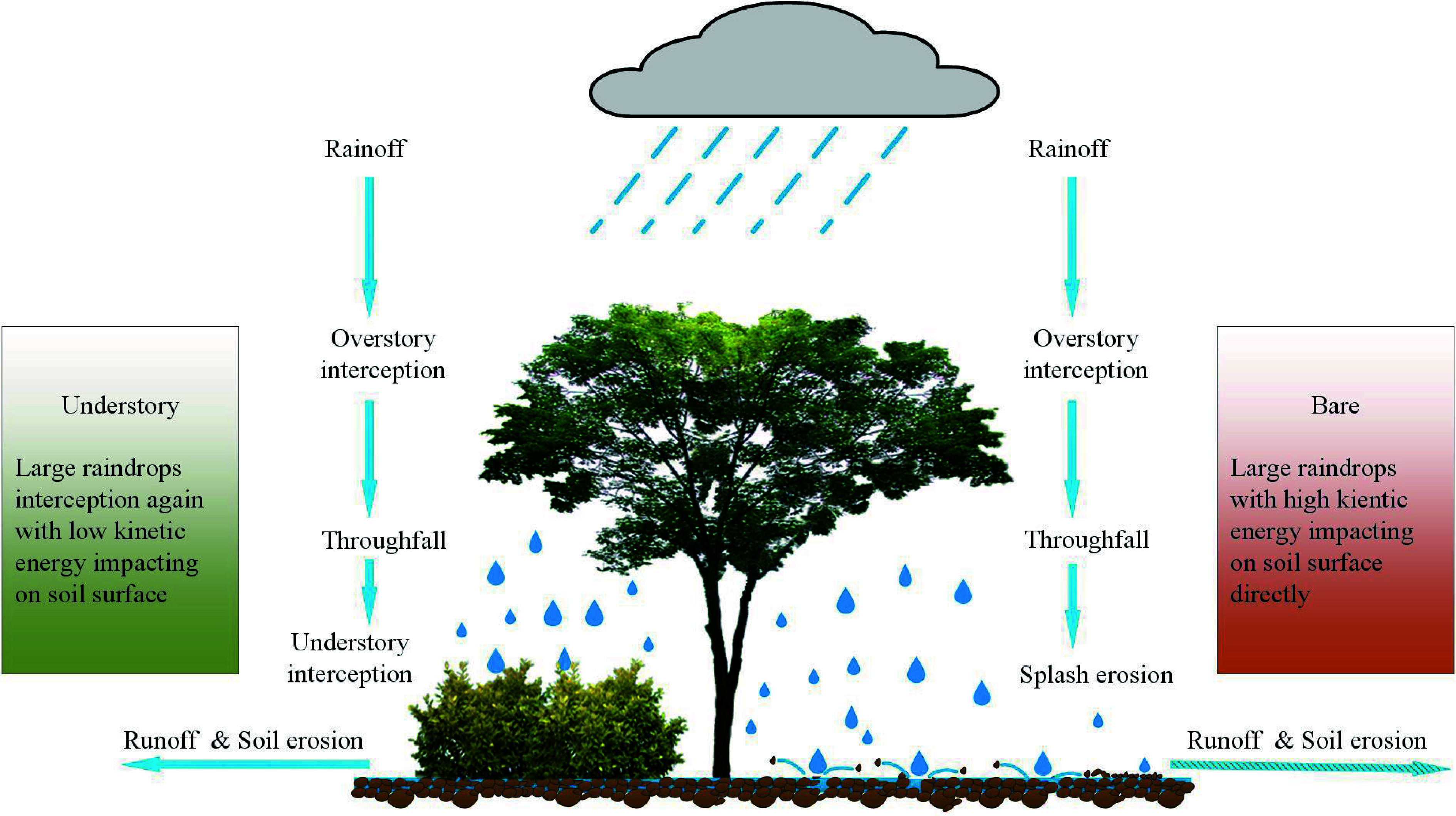
Schematic diagram of forest understory functioning on soil and water conservation. Rainfall is first intercepted by overstory, and then throughfall (large raindrops) directly impacts on the soil with high kinetic energy (splash erosion), if there is no second interception by understory vegetation. It would result in more water runoff and sediment erosion.

Soil and water conservation is one of the major functions of forest vegetation, including reducing the splash erosion of rainfall, weakening runoff and sediment erosion, and improving soil structure^[276,277]^. Although the overstory canopy intercepts the amount of rainfall that reaches the ground, it significantly increases the size diversity of raindrops. In the case of a high overstory-canopy forest with less understory vegetation and litter layer, these large raindrops (i.e., throughfall) would have a substantial impact on the soil surface, resulting in soil erosion (Fig. 9). Therefore, understory vegetation is essential in forest ecosystems as a protective barrier against soil erosion^[278−282]^. Here, we reviewed the importance of understory vegetation in soil and water conservation in both natural and plantation forests, aiming to provide more attention to the effect of understory vegetation on forest ecosystem function.

In natural forests, throughfall is redistributed by understory vegetation before finally reaching the ground. It minimizes the kinetic energy of throughfall by decreasing the falling height to protect the soil surface and its structure^[283]^. Moreover, understory vegetation modifies the amount of throughfall interception, and facilitates soil infiltration into the subsoil layer, ultimately reducing surface runoff and soil erosion^[284−287]^. Previous studies have also reported that soil erosion increases with a decreased understory vegetation in forests^[288]^. Artificial manipulation in a moso-bamboo forest shows that understory vegetation removal accelerates splash erosion and soil erosion rate^[289]^. In the evergreen broadleaved, Japanese cypress and cedar forests, understory removal increases runoff coefficients and the rate of soil erosion^[290]^. Similar results have also been observed in coniferous forests^[291,292]^. These consistent findings imply why natural forests without disturbance, generally have a solid function of soil and water conservation. However, the mechanism of understory vegetation in forest ecosystem's hydrological cycle needs to be further clarified, which is necessary to evaluate the role of understory vegetation in soil erosion modelling.

In reforestation and tree crop plantations, however^[171,293]^, understory vegetation is sparse compared to natural forests due to tillage management, fertilization, and herbicide applications. It increases the risk of soil and nutrient losses^[294]^. Thus, most of the tree plantations that are monoculture forests generally have a severe risk of soil erosion. A typical case is observed in the *Eucalypts* plantation in southern China^[295]^; due to the lack of understory vegetation under the tree canopy, the runoff coefficient and erosion rate are higher than those in mixed forests. Therefore, protecting or developing understory vegetation can alleviate soil erosion and nutrient loss in reforestation and tree crop plantations^[293,294,296]^. Understory vegetation should be retained as much as possible if its height is low enough to slow splash impact. For instance, intercropping legumes in an olive plantation reduces soil loss^[297]^. Splash erosion in rubber plantations is effectively controlled by constructing a rubber agroforestry system^[298]^. Surface runoff and soil loss are limited by promoting understory vegetation in teak plantations^[299]^. All highlight the importance of understory vegetation in soil and water conservation and suggest appropriate management in controlling runoff and soil erosion.

Although in recent years, more studies have been focusing on the effect of understory vegetation on soil and water conservation, we identified that many critical knowledge gaps still need to be filled. First, optimizing soil erosion modelling by integrating the function parameters of understory vegetation in forest hydrological cycle processes is necessary. Most interception loss models have focused on canopy components to accurately estimate soil erosion in forests, while interception loss from understory vegetation is largely ignored^[300]^. A complete interception model is essential for understanding the kinetic energy of rainfall splashes and soil erosion. Second, understory species have specific abilities in reducing runoff and soil erosion. Therefore, future research should focus on different types of understory vegetation in improving soil conditions to reduce soil erosion, such as increasing soil infiltration, enhancing soil aggregate stability, and improving soil resistance through roots. Finally, reducing soil erosion is essential for guaranteeing forest ecosystem function. A full assessment of different understory vegetation management practices is needed in diverse forest systems to achieve soil and water conservation, especially for tree plantations. An optimal management practice should be proposed to contribute to the sustainable development goals of human beings.

## Closing remarks

Understory vegetation as the 'darling' of forest ecosystems has received increasing attention. The present review highlighted recent advances and achievements in studies of understory vegetation, with a focus on its species characteristics and potential ecological effects. Topics ranged from forest biodiversity, ecosystem structure and functioning to ecological services. Even though understory vegetation plays a significant role in forest ecosystems, many problems remain to be addressed. In the context of pervasive global change, future research should focus on the plant‒soil relationship and above‒below feedback of understory vegetation and overstory tree interactions, which is driven by multiple climate drivers interacting with forest management practices at regional scales. It will be achieved by applications of cutting-edge methods, including isotope labeling techniques, high-throughput sequencing analysis, organic matter grouping, and the like. Additionally, more nondestructive approaches should be developed for monitoring and characterizing understory vegetation dynamics together with biodiversity ground-based surveys. Especially, 3D LiDAR scanning and hyperspectral scanning techniques feature the advantages of time savings, low cost, ease of operation, and high possibility for large-scale studies over extended periods. We hope that these new insights can better guide understory vegetation management to achieve a 'win‒win' situation of forest ecological and economic benefits.
